# A meta-analysis of the vascular-related safety profile and efficacy of α-adrenergic blockers for symptoms related to benign prostatic hyperplasia

**DOI:** 10.1111/j.1742-1241.2008.01880.x

**Published:** 2008-10

**Authors:** J C Nickel, S Sander, T D Moon

**Affiliations:** 1 Division of Urology, Queen’s UniversityKingston, Canada; 2 Boehringer Ingelheim Pharmaceuticals, Inc.Ridgefield, CT, USA; 3 Division of Urology, University of Wisconsin School of Medicine and Public HealthMadison, WI, USA

## Abstract

**Objectives:**

To evaluate the safety profile and efficacy of α1-adrenergic receptor blockers (A1Bs) currently prescribed for benign prostatic hyperplasia (BPH).

**Data sources:**

A systematic literature search of MEDLINE, the Cochrane Database and the Food and Drug Administration Web site through December 2006 identified double-blinded, prospective, placebo-controlled trials, evaluating agents commercially available by prescription for the symptomatic treatment of BPH.

**Review methods:**

Data were reviewed by two investigators with the use of a standardised data abstraction form. Studies were evaluated for methodological quality using the Jadad scale. Studies with a score of < 3 were considered of weaker methodology.

**Results:**

Of 2389 potential citations, 25 were usable for evaluation of safety data, 26 for efficacy. A1B use was associated with a statistically significant increase in the odds of developing a vascular-related event [odds ratio (OR) 2.54; 95% confidence interval (CI): 2.00–3.24; p < 0.0001]. The odds of developing a vascular-related adverse event were: alfuzosin, OR 1.66, 95% CI: 1.17–2.36; terazosin, OR 3.71, 95% CI: 2.48–5.53; doxazosin, OR 3.32, 95% CI: 2.10–5.23 and tamsulosin, OR 1.42, 95% CI: 0.99–2.05. A1Bs increased *Q*_max_ by 1.32 ml/min (95% CI: 1.07–1.57) compared with placebo. Difference from placebo in American Urological Association symptom index/International Prostate Symptom Score was −1.92 points (95% CI: −2.71 to −1.14).

**Conclusions:**

Alfuzosin, terazosin and doxazosin showed a statistically significant increased risk of developing vascular-related events compared with placebo. Tamsulosin showed a numerical increase that was not statistically significant. All agents significantly improved *Q*_max_ and symptom signs compared with placebo.

**What’s known**

The four most frequently prescribed α1-adrenergic receptor blockers (A1Bs) (alfuzosin, terazosin, doxazosin including the GITS formulation and tamsulosin) are all effective in relieving the symptoms of benign prostatic hyperplasia (BPH). However, it is also known that these four agents vary in their subtype selectivity and are associated with differing side effect profiles. Meta-analysis to determine the safety-related adverse event profile of the A1Bs has been performed in the past, but newer treatment practices and new formulations of older agents may invalidate these prior analyses.

**What’s new**

This meta-analysis shows that the A1Bs alfuzosin, terazosin, doxazosin and doxazosin GITS statistically significantly increased the risk of developing vascular-related adverse events compared with placebo. Tamsulosin, an A1B with subtype selectivity to the α-1_A_ and α-1_D_, showed a numerical increase in risk that was not statistically significant compared with placebo. All agents significantly improved *Q*_max_ and symptom score compared with placebo.

## Introduction

Benign prostatic hyperplasia (BPH) is a highly prevalent disorder that affects approximately 50% of men aged 65 years and older and is associated with lower urinary tract symptoms (LUTS) (1). The cluster of BPH-related LUTS, which include nocturia, frequency, urgency, hesitancy, intermittency and incomplete emptying, can negatively impact health-related quality of life (2,3). BPH can also lead to more serious complications, such as acute urinary retention, urinary tract infections, long-term renal insufficiency, and haematuria (4). The initial assessment of BPH/LUTS involves symptom assessment which ideally includes administration of the seven-item American Urological Association symptom index (AUA-SI), which evaluates the presence and severity of the main components of LUTS (2). These seven questions have been internationally adopted, with the addition of an eighth question related to bother, as the International Prostate Symptom Score (IPSS) (2).

The AUA recommends α1-adrenergic receptor blockers (A1Bs) as safe and efficacious pharmacologic treatment options for patients suffering from BPH (2). A1Bs block the adrenergic receptors, which are abundant in the smooth muscle of the prostate and bladder, produces a reduction in smooth muscle tone (5). Of the three A1B subtypes (α1_A_, α1_B_ and α1_D_), α1_A_ is seen as the primary regulator of smooth muscle tone in the bladder neck and prostate (6,7). In contrast, the α1_B_ subtype regulates blood pressure via arterial smooth muscle relaxation, while the α1_D_ subtype is associated with contraction of the bladder muscle as well as sacral spinal cord innervation (6–8).

The four most frequently prescribed A1Bs – terazosin, doxazosin [also available as doxazosin gastrointestinal therapeutic system (GITS)], alfuzosin and tamsulosin – vary in their subtype selectivity and are associated with differing side effect profiles (2). Because α-blockers cause vasodilation, vascular-related adverse events take the form of dizziness, presyncope or syncope. These symptoms can be life threatening, particularly in an older patient population. Terazosin and doxazosin, originally developed as antihypertensive drugs, are non-subtype-selective A1Bs, and both are associated with a larger number of vasodilatory side effects than either tamsulosin or alfuzosin (9–12). Both terazosin and doxazosin require titration in order to reduce the risk of vasodilatory side effects. While alfuzosin is also a non-subtype-selective A1B, it is considered uroselective; it is associated with fewer vasodilatory adverse events and does not require titration (13–15). Tamsulosin differs from the other A1Bs in that it is selective for the α1_A_ and α1_D_ subtypes (16). Tamsulosin is associated with a low incidence of vasodilatory side effects and does not require titration (17,18).

The present meta-analysis was conducted to assess vascular-related adverse events and efficacy among four available A1Bs used to treat BPH/LUTS. Because vascular-related adverse events constitute the only category of BPH treatment-related adverse events that have the potential to be life threatening, this study focuses on these events. Although meta-analyses of this nature have been done in the past, such studies may no longer accurately account for current BPH treatment practices. The recent development of newer dosing formulations (including extended-release formulations) of the available BPH medications may provide superior safety compared with the earlier selection of formulations, and while earlier studies analyzed each respective compound taken as a whole (i.e. without regard to differences between formulations), this study evaluates only the specific formulation and doses of each BPH medication currently used.

## Methods

### Study Selection

Studies evaluated in this meta-analysis were derived from a literature search of MEDLINE from 1966 through December 2006, the Cochrane Database, and the Food and Drug Administration (FDA) web site. An optimally sensitive search strategy was employed to identify randomised trials (19). Additionally, a manual search of references from identified clinical trials and review articles, as well as relevant presentations pertaining to BPH, were performed. Key words included ‘benign prostatic hyperplasia’, ‘BPH’, ‘alpha-1 adrenergic receptor antagonist’, ‘terazosin’, ‘doxazosin’, ‘tamsulosin’, ‘alfuzosin’, ‘safety’, ‘adverse event’ and ‘efficacy’. All searches were limited to clinical trials in human subjects and reports published in English. Inclusion criteria for studies in this analysis required that they be double-blinded, prospective, placebo-controlled trials evaluating agents commercially available by prescription for the symptomatic treatment of BPH. Trials that were performed with immediate-release alfuzosin were excluded, as that formulation is not available. Alfuzosin trials were limited to those that use the current controlled-release formulation. Included trials also had to evaluate one of the meta-analysis outcome measures described below.

### Outcome measures

The primary outcome measure was the odds of experiencing a vascular-related adverse event among A1Bs, defined as the occurrence of one of the following: dizziness, hypotension or syncope. Other outcomes included: (i) adverse events potentially related to the effect of A1Bs on peripheral vasculature including asthenia, fatigue and headache, (ii) efficacy of A1Bs based on change from baseline of maximum urinary flow rate (*Q*_max_) and change from baseline of AUA-SI or IPSS. Only those events reported in the trials as adverse events, and not actively sought after, were evaluated (e.g. patients who met a predetermined change in systolic blood pressure upon standing were not considered for this analysis as having hypotension, whereas patients who reported hypotension outside the physician’s office were).

### Validity assessment and data abstraction

All data were reviewed by two investigators with the use of a standardised data abstraction form. The included studies were evaluated for methodological quality using the Jadad scale (20). Studies with a score of < 3 were considered of weaker methodology. For each study, the following data were collected: authorship, year of publication, mean age, length of treatment, entry criteria, prostate size, average dose, appropriate use of randomisation, random allocation concealment, masking of treatment allocation and blinding, sample size, total per cent of patients discontinued, per cent of patients withdrawn because of adverse event, specific A1Bs and dose, type of adverse event and raw incidence data or odds ratio (OR) and 95% confidence interval (CI), change in IPSS/AUA and *Q*_max_ and weighted mean difference (WMD) from placebo.

### Statistical analysis

For the safety evaluation, outcome measures were dichotomous and expressed in terms of pooled OR relative to placebo with accompanying 95% CIs. Analysis of A1B safety and efficacy was based on the intention-to-treat population for each given trial. Analyses were conducted using StatsDirect statistical software version 2.4.5 (Stats-Direct Ltd, Cheshire, UK) using random-effects model (DerSimonian and Laird methodology). p < 0.05 was considered statistically significant. Statistical heterogeneity of the primary end-point was measured using the Cochrane’s *Q* statistic (p < 0.1 was considered to represent heterogeneity).

To establish the effect of clinical heterogeneity between studies on our meta-analysis’ conclusions, subgroup analysis was conducted. As the effect of A1Bs may vary, the impact of individual A1B use on the odds of developing a vascular-related adverse event was evaluated.

For the efficacy evaluation, change in *Q*_max_ and change in symptom scores were expressed as WMD from baseline with accompanying 95% CIs. Changes from baseline were compared between treatment and placebo and expressed as the difference (treatment − control) of the changes (baseline − follow-up) in these mean values. In studies in which the variance of changes was not reported directly, variances were calculated from CIs, *t*-statistics, p-values or individual variances for intervention and control groups (parallel trials). For trials in which variance of paired differences was reported separately for each group, a pooled variance for net change was calculated by standard methods. When the variance for paired differences was not reported, it was calculated from variances at baseline and at the end of follow-up. A correlation coefficient of 0.5 between initial and final values was assumed (21). Additionally, equal variances were assumed during the trial and between intervention and control groups.

Studies evaluating tamsulosin used a dose of 0.4 mg, and studies evaluating alfuzosin used a dose of 10 mg daily. The evaluated doses for doxazosin ranged from 2–8 mg/day, for doxazosin GITS from 4 to 8 mg/day, and for terazosin from 1 to 10 mg/day. Patients on terazosin or doxazosin were either titrated based on response or randomly assigned a predetermined fixed dose.

Publication bias was assessed using several methods. Visual inspection of a funnel plot for vascular-related end-points was performed. A funnel plot provides a visual representation of each study included in the meta-analysis plotted by its effect size on the horizontal axis and variance on the vertical axis. When publication bias is not present, the funnel plot resembles an inverted funnel, with less precise studies having greater variance scattered at the bottom to either side of the more precise studies. If publication bias is present in a meta-analysis, the plot does not appear as an inverted, symmetrical funnel. The Egger’s weighted regression method was also used to assess publication bias (p< 0.05 was considered representative of statistically significant publication bias) (22).

## Results

### Study characteristics

**Figure 1:**
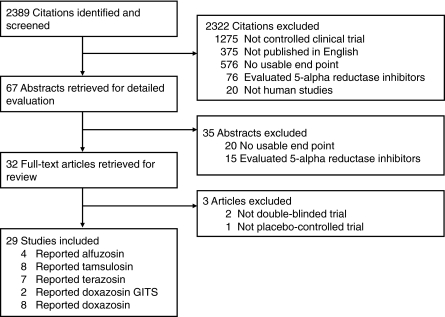
Flow chart for study selection

Our initial search yielded 2389 potential citations, of which 2360 were excluded for reasons presented in Figure 1. Table 1 provides an overview of the composite baseline characteristics of all trials included in the meta-analyses, including entry criteria, treatment dosages, discontinuation rates and Jadad scores (9–11,17,18,23–45). With regard to entry criteria, there was an overall similarity between A1B trials. Patient enrolment for A1B trials ranged from 30 to 1053 patients, and the mean age ranged from 58 to 68 years. A total of 25 studies evaluated safety data and 26 studies reported efficacy data among A1Bs. The studies evaluated alfuzosin (*n* = 4), tamsulosin (*n* = 8), terazosin (*n* = 7), doxazosin GITS (*n* = 2) and doxazosin (*n* = 8). The treatment duration of the A1B trials most commonly ranged from 4 to 24 weeks, although several trials lasted 1 or 2 years and in one case lasted for 4.5 years.

**Table 1:** Characteristics of clinical trials included in the meta-analysis

	*n*	Mean age (years)	Length of treatment	Entry criteria	Prostate volume (ml)/ size (g)	Average dose (mg/day)	% Discontinued (tx/placebo)	% Withdrawn due to AE (tx/placebo)	Jadad score
*A1Bs*									
**Alfuzosin**									
van Kerrebroecket al. (AFORTI) (23)	154	64.9	12 weeks	> 50 years, IPSS ≥ 13, *Q*_max_ = 5–12, VV ≥ 150, PRV ≤ 350	–	10	11.2/6.5	–	3
Roehrborn (ALFUS) (24)	177	64.3	12 weeks	> 50 years, IPSS ≥ 13, *Q*_max_ = 5–12, VV ≥ 150, PRV ≤ 350,QOL index ≥ 3 (0–6 scale), PSA ≤ 10 ng/ml	40.2/–	10	11/11	4.5/2.2	3
ALFOTAM – alfuzosin (25)	154	64.6	12 weeks	> 50 years, IPSS ≥ 13, *Q*_max_ = 5–12, VV ≥ 150, PRV < 350, nocturia ≥ 2	–	10	5.8/7.8	2.6/3.2	4
Roehrborn (ALTESS) (26)	759	66.4	2 years	> 55 years, IPSS 13, *Q*_max_ = 5–12, VV ≥ 150, PRV ≤ 350, prostate ≥ 30 g, PSA 1.4–10	46.9/–	10	30.3/37.1	9.4/8.1	4
**Tamsulosin**									
Kawabe et al. (27)	59	68 (allpatients in study	4 weeks	43–84 years, mild BPH, *Q*_max_ < 15 or *Q*_ave_ < 7.5, VV ≥ 50, PR ≥ 30	–	0.4	–	–	4
Abrams et al. (28)	198	63.3	12 weeks	≥ 45 years, Boyarsky > 6, *Q*_max_ = 4–12, VV ≥ 120, PRV ≤ 400	≈33/≈34	0.4	7/6	4/3	5
Chapple et al. (29)	382	63.6	12 weeks	≥ 45 years, Boyarsky > 6, *Q*_max_ = 4–12, VV ≥ 120, PRV ≤ 400	–	0.4	8/7	4/4	5
ALFOTAM – tamsulosin (25)	158	63.9	12 weeks	> 50 years, IPSS ≥ 13, *Q*_max_ = 5–12, VV ≥ 150, PRV < 350, nocturia ≥ 2	–	0.4	6.0/7.8	3.8/3.2	4
Abrams et al. (30)	30	≈65 (allpatients in study)	4 weeks	50–85 years, *Q*_max_ < 15, VV > 100,PRV ≤ 400, urethralresistance (detrusor pressure/*Q*_max_^2^)≥ 0.5	32.5/– (allpatients in study)	0.4	–	3.3/7.1	5
Lepor (17,18)	254	*	13 weeks	≥ 45 years, AUA-SI ≥ 13, *Q*_max_ = 4–15, PRV < 400, DBP ≥ 65, PR ≤ 120		0.4	16/19	7/9	5
Narayan and Tewari (31)	248	58 (all patientsin study)	13 weeks	≥ 45 years,moderate-to-severesigns and Sx of BPH	–	0.4	–	–	4
Chapple et al. (32)	1065	61.3	12 weeks	≥ 45 years, IPSS ≥ 13, *Q*_max_ = 4–12, VV ≥ 120	43–45	0.4	11/6	0/0	4
**Terazosin**									
Lepor and Laddu (33)	192	–	12 weeks	Boyarsky ≥ 1 on≥ 2 obstructive Sx, *Q*_max_ = 5–12,VV > 150, PRV ≤ 200	–	6	–	–	3
Lepor et al. (34)	216	61.8	12 weeks	44–77 years, Boyarsky ≥ 1 on≥ 2 obstructive Sx, ensp;*Q*_max_ = 5–12, VV > 150, DBP < 115	–/≈36.7	2/5/10	16.2/18.8	6.9/4.3	5
Lloyd 1992 (35)	66	65.7	8 weeks	> 45 years, 2 obstructive Sx, *Q*_max_ ≤ 12, VV ≥ 100, PRV < 150, DBP ≤ 115	–	6	–	6/0	3
Brawer et al. (36)	81	64	24 weeks	≥ 45 years, Boyarsky ≥ 1 on≥ 2 obstructive Sx, *Q*_max_ = 5–12	–	7	–	14.8/8.9	5
Roehrborn et al. (11)	1053	65.7	1 year	≥ 55 years, AUA-SI ≥ 13, AUA-BS ≥ 8, *Q*_max_ = 5–12, VV ≥ 150, PRV ≤ 350	–	5/10	38/46	16/11.1	3
Elhilali et al. (37)	80	64.1	24 weeks	50–80 years, Boyarsky ≥ 1 on≥ 2 obstructive Sx, *Q*_max_ ≤ 15,VV > 150, PRV < 250, DBP ≤ 115	–	1–10	–	8.8/4.9	4
Lepor et al. (38)	305	65.6	1 year	45–80 years, AUA-SI ≥ 8, *Q*_max_ = 4–15, VV ≥ 125, PRV < 300, BP ≥ 90/70	37.5/–	5/10	16/16.7	5.9/1.6	5
**Doxazosin GITS**									
Roehrborn et al.(39) (Dox GITS)	108	63.5	2 weeks	50–80 years, IPSS ≥ 12, *Q*_max_ = 5–15, VV ≥ 150, PRV ≤ 200, BP ≥ 90/60,enlarged prostate, PSA < 4 (PSA 4–10if malignancy ruled out by 2 tests)	–	4	–	4.6/1	5
Andersen et al.(40) (Dox GITS)	317	64.9	13 weeks	50–80 years, IPSS ≥ 12, *Q*_max_ = 5–15, VV ≥ 150, PSA ≤ 10	–	6.2	6.9/5.1	3.5/0.6	4
**Doxazosin**									
Janknegt and Chapple (41)(The Netherlands)	50	–	5 weeks	≈ 50–80 years, *Q*max ≤ 15	–	2	–	–	3
Christensen et al. (42)	52	66.7	9 weeks	Moderate-to-severe Sx of BPH	–	4	7.7/10.4	0/4.2	3
Chapple et al. (43)	67	67	12 weeks	*Q*_max_ < 15, VV > 150, PRV < 200, Sx of BOO	–	4	10.4/7.4	3/0	3
Fawzy et al. (44)	50	62.1	14 weeks	≥ 45 years, AUA-SI ≥ 10,*Q*_max_ = 5–15, VV 125–500,PRV < 250, DBP < 90	–	7	22/22.9	14/2.1	3
Gillenwater et al. (45) (htn)	199	64	14 weeks	≥ 45 years, mild-to-moderate hypertension,*Q*_max_ = 5–15, VV 150–500,PRV < 200, DBP 90–114, frequency ≥ 4, nocturia > 2, PSA ≤ 10	–	7	34.7/36.7	11.1/4.1	3
Andersen et al. (40) (dox)	322	65.3	13 weeks	50–80 years, IPSS ≥ 12, *Q*_max_ = 5–15, VV ≥ 150, PSA ≤ 10	–	5.7	11.8/5.1	6.2/0.6	4
Kirby et al. (PREDICT) (10)	275	63	1 year	50–80 years, IPSS ≥ 12, *Q*_max_ = 5–15, VV ≥ 150, enlarged prostate,PRV ≤ 200, DBP ≥ 95/60	–/36.3	6.4	28.4/28.1	11.6/11.1	4
McConnell et al. (MTOPS) (9)	756	62.7	4.5 year	≥ 50 years, AUA 8–30, *Q*_max_ = 4–15, VV ≥ 125, DBP ≥ 90/70, PSA ≤ 10	36.9/–	4/8	27/–	–	5

* Mean age is not provided, but patients in tamsulosin group were reported as being significantly younger (p = 0.005). A1B, α1-adrenergic receptor blockers; AUA-BS, American Urological Association bother score; AUA-SI, American Urological Association symptom index; BOO, bladder outlet obstruction; BP, blood pressure; BPH, benign prostatic hyperplasia; DBP, diastolic blood pressure; IPSS, International Prostate Symptom Score; PR, pulse rate; PRV, postvoiding residual volume; PSA, prostate-specific antigen; *Q*_ave_, average urinary flow rate; *Q*_max_, maximum urinary flow rate; QOL, quality of life; Sx, symptoms; Tx, treatment; VV, voided volume.

### Quantitative data synthesis

#### Safety

**Figure 2:**
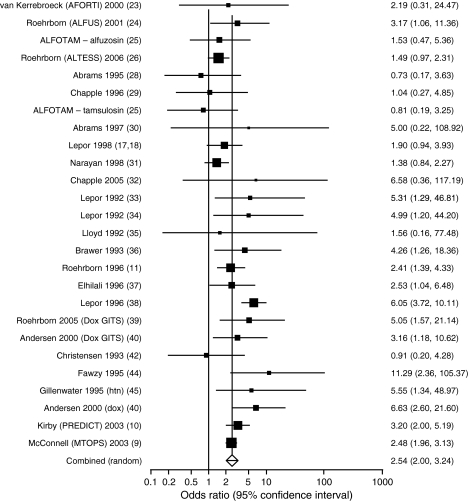
The effect of α1-adrenergic receptor blockers on vascular-related adverse events. Sizes of the data markers are indicative of the relative weight of each study. The bar is representative of the 95% confidence interval

**Figure 3:**
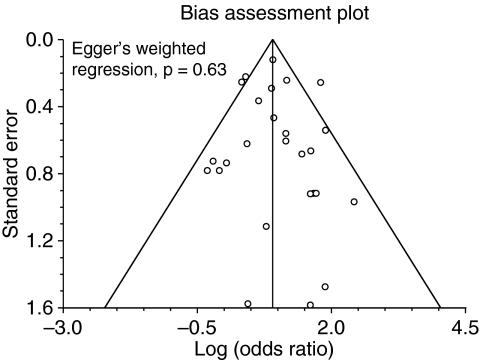
Funnel plot of safety analysis of α1-adrenergic receptor. Plots represent 25 studies evaluating vascular-related event among α1-adrenergic receptor blockers

Figure 2 presents ORs and 95% CIs for each trial and the overall combined primary end-point of the vascular-related adverse event. A1Bs were associated with a statistically significant increase in the odds of developing a vascular-related adverse event relative to placebo. (OR 2.54; 95% CI: 2.00–3.23; p< 0.0001). Heterogeneity could not be ruled out through Cochrane’s Q statistic (p = 0.011). Publication bias was not evident with review of the funnel plot (Figure 3) or Egger’s weighted regression (p = 0.63).

**Figure 4:**
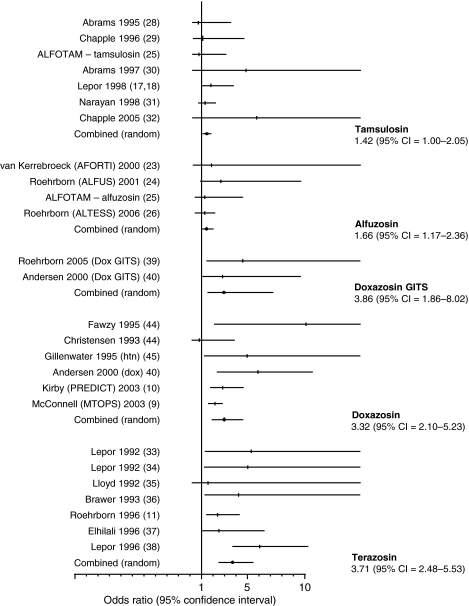
Odds of developing a vascular-related adverse event while on specific α1-adrenergic receptor blockers. Sizes of the data markers are indicative of the relative weight of each study. The bar is representative of the 95% confidence interval

Subgroup analysis was conducted, and the results are depicted in Figure 4 and Table 2. When A1Bs were evaluated individually, differences in vascular-related adverse events were observed. There was a significantly higher odds of developing the primary composite end-point relative to placebo for alfuzosin (p = 0.005), terazosin (p< 0.0001), doxazosin (p< 0.0001) and doxazosin GITS (p< 0.0001). The odds of developing a vascular event was higher with tamsulosin relative to placebo, but the difference was not statistically significant (p *=*0.053). Statistical heterogeneity was not present for alfuzosin, terazosin and tamsulosin (*Q*-statistic p >0.1); however, statistical heterogeneity could not be ruled out for doxazosin (*Q*-statistic p *=*0.039).

**Table 2:** Safety analysis of α1-adrenergic receptor blockers

	Alfuzosin (*n* = 2475)			Tamsulosin (*n* = 3004)			Terazosin (*n* = 3701)			Doxazosin (*n* = 2249)			Doxazosin GITS (*n* = 686)		
					
	OR	95% CI	p-value	OR	95% CI	p-value	OR	95% CI	p-value	OR	95% CI	p-value	OR	95% CI	p-value
Dizziness	**1.49**	**1.02, 2.17**	**0.040**	1.35	0.97, 1.88	0.075	**3.06**	**2.22, 4.20**	**< 0.0001**	**2.890**	**1.804, 4.631**	**< 0.0001**	**4.196**	**1.748, 10.074**	**0.001**
Hypotension	2.44	0.86, 6.79	0.095	1.13	0.17, 7.54	0.897	**5.36**	**2.61, 11.00**	**< 0.0001**	**2.525**	**1.578, 4.041**	**< 0.0001**	2.608	0.706, 9.630	0.150
Headache	1.38	0.83, 2.29	0.209	0.97	0.72, 1.30	0.834	1.07	0.65, 1.75	0.800	0.992	0.500, 1.967	0.982	1.456	0.683, 3.100	0.330
Asthenia/fatigue	1.42	0.79, 2.57	0.240	1.38	0.87, 2.19	0.170	**2.42**	**1.79, 3.28**	**< 0.0001**	**2.434**	**1.861, 3.184**	**< 0.0001**	3.168	0.908, 11.049	0.071
Syncope	2.62	0.61, 11.32	0.196	0.77	0.16, 3.73	0.740	1.96	0.41, 9.37	0.400	1.963	0.177, 21.781	0.985	–		
Dizziness, hypotensionor syncope	**1.66**	**1.17, 2.36**	**0.005**	1.42	0.99, 2.05	0.053	**3.71**	**2.48, 5.53**	**< 0.0001**	**3.320**	**2.100, 5.230**	**< 0.0001**	**3.860**	**1.860, 8.020**	**< 0.0001**

CI, confidence interval; OR, odds ratio; GITS, gastrointestinal therapeutic system. Bold values indicate statistical significance relative to placebo.

#### Efficacy

**Figure 5:**
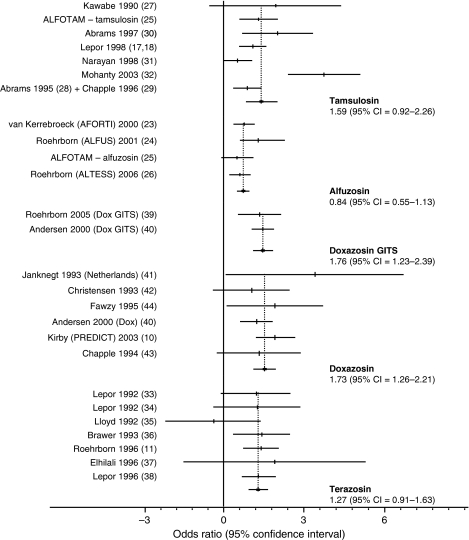
Weighted mean difference of α1-adrenergic receptor blockers in maximum urinary flow rate from placebo

*Q*_max_ for all A1Bs improved by 1.32 ml/min (95% CI: 1.07–1.57; p< 0.0001). The WMD in AUA-SI/IPSS for all A1Bs was −1.92 points (95% CI, −2.71 to −1.14); p< 0.0001). Individual differences from placebo in efficacy are reported in Table 3, and individual A1B results for *Q*_max_ are presented in Figure 5.

**Table 3:** Efficacy analysis of α1-adrenergic receptor blockers

	Alfuzosin			Tamsulosin			Terazosin			Doxazosin			Doxazosin GITS		
					
	WMD	95% CI	p-value	WMD	95% CI	p-value	WMD	95% CI	p-value	WMD	95% CI	p-value	WMD	95% CI	p-value
Change in IPSS/AUA	−1.67	−2.11, −1.23	< 0.0001	−3.06	−4.79, −1.33	0.0005	−3.40*	−4.29, −2.51	< 0.0001	−2.49	−3.20, −1.78	< 0.0001	−2.16	−2.99, −1.33	< 0.0001
Change in Qmax	0.84	0.55, 1.13	< 0.0001	1.59	0.92, 2.26	< 0.0001	1.27	0.91, 1.63	< 0.0001	1.73	1.26, 2.21	< 0.0001	1.76	1.13, 2.39	< 0.0001

* Only based on one trial (38). WMD, weighted mean difference; AUA, American Urological Association; CI, confidence interval; IPSS, International Prostate Symptom Score; OR, odds ratio; *Q*_max_, maximum urinary flow rate; GITS, gastrointestinal therapeutic system.

## Discussion

The present meta-analysis of A1Bs in the treatment of BPH and its related symptoms is, to our knowledge, the most complete of its kind to date. The results demonstrate that the use of A1Bs in BPH treatment confers an added risk of vascular-related adverse events compared with placebo. The exception to this finding was that tamsulosin, although associated with a trend toward greater odds of experiencing a vascular-related adverse event, was not statistically significantly different from placebo.

The occurrence of vasodilatory side effects among patients using A1Bs for the treatment of BPH/LUTS may be related to the specific selectivity profile for α-adrenergic receptor subtypes of each individual agent (8). Of the three known α-adrenergic receptors –α1_A_, α1_B_ and α1_D_–α1_A_ is predominant in the prostate, while the α1_B_ subtype is localised in the peripheral vasculature, and the α1_D_-receptor subtype is expressed in the bladder and spinal cord (6–8). Terazosin and doxazosin are long-acting, non-subtype-selective A1Bs, both of which were initially developed and marketed as antihypertensive agents. This non-selectivity and propensity to induce vascular-related effects has been demonstrated, in that both drugs, when used at therapeutic levels, are associated with an increased risk for hypotension and dizziness (9,12,38). Indeed, the results of this meta-analysis reflect the heightened odds for experiencing both of these side effects, as well as increased risk for vascular-related side effects overall, with terazosin and doxazosin treatment. Doxazosin GITS is a controlled release formulation of doxazosin that reduces the peak-to-trough ratio minimising the need for titration (40). Nevertheless, the odds of experiencing a vascular event are similar to those of non-subtype-selective A1Bs. Alfuzosin is also non-subtype-selective. This may account for the observed increase in the odds for an adverse vascular event with alfuzosin treatment (7,8). Tamsulosin is much more selective at α1_A_ and α1_D_ receptors than at α1_B_ receptors (46). Tamsulosin’s low risk of vasodilatory effects, as observed in the results of this meta-analysis, is likely the result of its subtype receptor selectivity. It is most likely because of these pharmacological differences of agents that statistical heterogeneity among trials could not be ruled out for any agent for the primary end-point. Upon subgroup analyses of trials of individual agents, however, statistical heterogeneity was not present for any agent except doxazosin.

While the safety aspect of this meta-analysis is limited to vascular-related adverse events because of their potentially life-threatening effects, it should be noted that A1Bs are associated with other kinds of adverse events, including those related to sexual function. This adverse event may be a significant differentiating factor that physicians use to determine which A1B treatment is optimal, particularly for younger sexually active men with BPH. Tamsulosin and terazosin are both associated with low but statistically significant increases in risk for abnormal ejaculation compared with placebo (11,47,48). Most studies of alfuzosin have observed no significant increase in risk of ejaculation disorder, although one comparative study of tamsulosin and alfuzosin found no significant difference between the two agents for risk of abnormal ejaculation (47–49).

In terms of treatment efficacy for BPH/LUTS, this meta-analysis found no differences in improving *Q*_max_ and AUA-SI/IPSS symptom scores among the different A1Bs compared with placebo. These results are consistent with earlier meta-analyses produced by the AUA Practice Guidelines Committee as well as by Djavan and Marberger (2,50). Taking this into consideration, the preference between A1Bs for the treatment of BPH/LUTS will be necessarily contingent, at least in part, upon the differing side effect profiles. This is particularly the case for vascular-related side effects, as BPH/LUTS disproportionately affects elderly patients who may be more susceptible than younger patients to such adverse events. Kaplan and Neutel underscored this point in a recent publication, in which they recommended that clinicians keep themselves knowledgeable about the latest clinical evidence for differential risk of vasodilatory side effects between the available A1Bs (51). They noted that the use of an A1B with the lowest risk of vascular-related adverse events is advisable for symptomatic older patients in order to ensure safe and effective BPH/LUTS treatment and to improve patient outcomes (51).

A common limitation in undertaking meta-analyses is the issue of publication bias, in which clinical trials with statistically significant results are published and those with undesirable results frequently are not (52). In conducting the present meta-analysis, an attempt was made to avoid publication bias by seeking out and including clinical trial data that have not been previously published in peer-reviewed journals (e.g. data from the FDA web site). Accordingly, publication bias was not present through visual inspection of the funnel plot or through Egger’s weighted regression. Lastly, this meta-analysis could not rule out heterogeneity through Cochrane’s *Q*-statistic. However, upon further assessment with subgroup analyses of trials of individual agents, statistical heterogeneity was not present for any agent except doxazosin.

## Conclusions

The present meta-analysis sought to evaluate the safety profile and efficacy of available pharmacologic agents for BPH and its related symptoms. Alfuzosin, terazosin, and doxazosin, and doxazosin GITS showed a statistically significant increased risk of developing vascular-related events compared with placebo, whereas tamsulosin showed a numerical increase that was not statistically significant. All agents significantly improved *Q*_max_ and symptom score compared with placebo.
